# Prevalence of metabolic syndrome in four phenotypes of PCOS and its relationship with androgenic components among Iranian women: A cross-sectional study

**DOI:** 10.18502/ijrm.v13i4.6888

**Published:** 2020-04-30

**Authors:** Narges Zaeemzadeh, Shahideh Jahanian Sadatmahalleh, Saeideh Ziaei, Anoshirvan Kazemnejad, Azadeh Mottaghi, Neda Mohamadzadeh, Maryam Movahedinejad

**Affiliations:** ^1^Department of Midwifery and Reproductive Health, Faculty of Medical Sciences, Tarbiat Modares University, Tehran, Iran.; ^2^Department of Biostatistics, Faculty of Medical Sciences, Tarbiat Modares University, Tehran, Iran.; ^3^Research Center for Prevention of Cardiovascular Diseases, Institute of Endocrinology and Metabolism, Iran University of Medical Sciences, Tehran, Iran.

**Keywords:** Polycystic ovary syndrome, Metabolic syndrome, Hyperandrogenism.

## Abstract

**Background:**

Polycystic ovary syndrome (PCOS) increases the risk of metabolic syndrome (MetS). Insulin resistance (IR) plays a major role in the pathophysiology of both PCOS and MetS.

**Objective:**

This study was designed to compare the prevalence of MetS among different phenotypes of PCOS and its relationship with androgenic components.

**Materials and Methods:**

182 participants eligible for this five-group comparative study were selected by convenience sampling method. They were classified according to the Rotterdam criteria: clinical and/or biochemical hyperandrogenism (H) + PCOS on ultrasound (P) + ovulation disorders (O) (n = 41), clinical and/or biochemical H + PCOS on P (n = 33), PCOS on P + O (n = 40), clinical and/or biochemical H + O (n = 37), and control (without PCOS) (n = 31). MetS was measured based on the National Cholesterol Education Program Adult Treatment Panel III criteria. Androgenic components included free-androgen-index (FAI), total-testosterone (TT) level and sex-hormone-binding-globulin (SHBG).

**Results:**

A significant difference was observed between the study groups in terms of MetS prevalence (p = 0.01). In phenotype H+P+O, there was a statistically significant positive association between TG and TT, and a significant negative association between SBP and DBP with SHBG. In phenotype O+P, WC was inversely associated with SHBG. In phenotype H+O, FBS and TG were positively associated with FAI but HDL was inversely associated with FAI. Moreover, WC and DBP were positively associated with TT in phenotype H+O. No associations were detected between MetS parameters and androgenic components in other PCOS subjects (phenotype H+P) and in the control group. TT was significantly higher in the PCOS group suffering from MetS (p = 0.04).

**Conclusion:**

According to the research results, hyperandrogenic components are potent predictors of metabolic disorders. Thus, we suggest that MetS screening is required for the prevention of MetS and its related complications in PCOS women.

## 1. Introduction

Polycystic ovary syndrome (PCOS) is the most frequent endocrinopathic disturbance among women of reproductive age (1). The prevalence of PCOS among Iranian women is 14.6% based on the Rotterdam definition (2). As specified by the Rotterdam criteria in 2003, PCOS is diagnosed when at least two of the following three constituents are present: clinical and/or biochemical hyperandrogenism (H), oligomenorrhea/amenorrhea (O), and polycystic sonographic view in ovaries (P) (3). Accordingly, the following four statuses are placed in PCOS description: classic form (H + P + O; presence of all the three criteria); absence of polycystic sonographic view in ovaries (H + O); absence of hyperandrogenism (O + P); and absence of menstrual cycle disorders (H + P). The metabolic abnormalities related to PCOS (specially hyperandrogenic phenotypes) are body fat increase, dyslipidemia, insulin resistance (IR), glucose intolerance, and hypertension (4). These PCOS complications elevate the risk of long-term health outcomes such as diabetes mellitus and cardiovascular diseases (5). On the other hand, IR is fundamental in PCOS pathophysiology (6).

Metabolic syndrome (MetS) is a collection of chronic metabolic derangements, which promotes the risk of serious diseases such as cardiovascular disturbances and diabetes (6, 7). These metabolic disorders comprise of dysglycemia, increased blood pressure, obesity (particularly abdominal adiposity), dyslipidemia as elevated levels of triglyceride, and decrease in high-density lipoprotein cholesterol (HDL) levels (7). Risk factors of the MetS include diabetes, a history of gestational diabetes, obesity, IR, and PCOS (8). Most of the metabolic disturbances of PCOS patients overlap with the components of MetS (6).

Hyperandrogenism (biochemical) include laboratory evidence of hyperandrogenemia (increase in serum androgenic components: total testosterone (TT) and free androgen index (FAI)) (9). Hyperandrogenism (HA), which accompanies PCOS frequently, is associated with IR (10). Some investigators represented HA as a key factor in IR (4). HA could be linked with some IR-related complications such as MetS (11). Some studies demonstrated that HA contributed in metabolic profile changes (4). To the best of our knowledge, there are very few studies that have compared the prevalence of MetS in two or three prevalent phenotypes of PCOS (8, 12). There is a scarcity of data concerning the MetS prevalence in four phenotypes of PCOS (13). Other studies have addressed the evaluation of MetS components among the phenotypes of PCOS (14, 15). Furthermore, there are lots of controversial data obtained from various studies in this context (14, 16-19).

Therefore, this study was designed to compare the frequency of MetS and the association between the components of MetS with androgenic components (TT, FAI, and sex hormone-binding globulin (SHBG)) among different phenotypes of PCOS in Iranian women. To our knowledge, this is the first study that has addressed the relationship of MetS with androgenic components among Iranian PCOS women.

## 2. Materials and Methods

This five groups comparative study (four phenotypes of PCOS as H + P + O, H + P, O + P, H + O, and one control group without PCOS) was designed as a cross-sectional study. Prior to conducting the study, a sample size calculation was performed using the correlation between serum androgenic indices and MetS components' levels obtained in our pilot study. The minimum correlation coefficient obtained was 0.5 (based on the calculated correlation between TT and triglycerides (TG)). The sample size was calculated based on the following parameters: study power = 80%, confidence interval = 95%. Thus, the appropriate sample size was calculated at at least 27 for each phenotype of PCOS and the control group. Eventually, at least 31 women were recruited in each group to allow the loss to follow-up. The present comparative study was accomplished from October 2014 to September 2015 in Tehran, Iran. Convenience sampling method was used for the recruitment of the study subjects. Assessment of eligibility for participation and recruitment of the participants were based upon the inclusion and exclusion criteria.

The inclusion criteria were age 18-40 yr, non-pregnant, Iranian race, and no medication with hormones known to influence serum androgen levels, including anti-androgenic drugs and oral contraceptive pills during three months before the study. Those participants who were currently taking antihypertensive, lipid-lowering drugs, and insulin-sensitizing and glucose-reducing agents (due to the high glucose level in their blood) were considered to have hypertension, hyperlipidemia, and hyperglycemia, respectively, and they were excluded from the study. Due to the drug effect on the results, we should not include the PCOS patients who were consuming metformin due to PCOS (not because of the high blood glucose level). Those participants who were not eligible for the study such as patients with thyroid dysfunction, abnormal prolactin levels, congenital adrenal hyperplasia (CAH), Cushing's syndrome (CS), androgen-secreting tumors, and diagnosed CVD, as well as those taking oral contraceptives and anti-androgenic drugs were excluded from this study.

Regarding the rarity of some phenotypes of PCOS (especially H + P), more than 108 women were required to be screened so that we could complete the sample size of at least 27 in the rare subgroups. At the end of the data collection, 197 participants were entered into the study. Of these, 15 participants were considered as withdrawn because of failure to follow-up: (H + P + O, n = 4), (H + P, n = 3), (O + P, n = 2), (H + O, n = 3), and (control, n = 3). Eventually, the number of participants in each subgroup of PCOS and control group was as follows: (H + P + O, n = 41), (H + P, n = 33), (O + P, n = 40), (H + O, n = 37), and (control, n = 31).

In total, 182 participants were recruited among the eligible women referred to the Department of Obstetrics and Gynecology at two hospitals and one clinic. The participants were classified into five groups - one control group and four different phenotypes of PCOS. The control group included 31 non-hirsute women (without clinical evidence of hyperandrogenism) with regular menstrual cycles (without anovulation) whose hormonal and sonographic assessment results were negative in terms of PCOS as well. These women had been selected from among university students who are residents in Tehran, healthy companions of the patients (not their sister) and patients referred to the clinic for checkup, pap test, vaginitis treatment, and other irrelevant reasons and without any impact on the study results. These participants had no history of consumption of medications related to PCOS and MetS. The socioeconomic status was assessed by checking the remaining income of the participant after deducting expenses per person in a month. The economic situation was divided into three levels: first level (weak), second level (average), and third level (good). Physical activity was classified into three levels according to the frequency of exercise for at least 20 min per wk: none, 1-2 times/wk, and ≥ 3 times/wk.

We described PCOS using the Rotterdam criteria by the existence of at least two of the following three: ovulation disorders (O), clinical and/or biochemical hyperandrogenism (H), and PCOS on ultrasound (P). At the initial examination, the survey of medical history, general checkup (anthropometric measurements and blood pressure), and classification of PCOS patients were carried out for all participants based on the Rotterdam criteria. At first, all participants were questioned about the regulation of the menstrual cycle and they were subjected to clinical examination in order to evaluate hirsutism based on the Ferriman-Gallwey score (FG-score). If both of these factors were normal in a participant, she was selected as a candidate for the control group (if the results of ultrasound and serum hormonal tests were also normal in terms of PCOS, these participants were confirmed as control group candidates). All participants were referred to an abdominal ultrasound and hormonal test after the initial evaluation and clinical examination. The women were divided into four PCOS subgroups based upon the FG-score (hirsutism; clinical HA), serum androgen measurement (biochemical HA), presence or absence of PCOS sonographic view in ultrasound evaluation, and menstrual irregularities (amenorrhea/oligomenorrhea).

### Clinical and biochemical measurements 

Anthropometric measurements, which were performed for all participants in this study, included body weight, height, and waist circumference measurements. Height and weight were scaled with the subjects in light clothes and without shoes. Waist circumference was evaluated using a flexible tape at the midline between the lower rib border and the curved superior border of the ilium (at the level of the umbilicus) at the end of normal exhalation, whereas the participants were in the standing position. BMI was computed based on the World Health Organization (WHO) guidelines (5). The calculation formula was weight (kg) divided by height squared (m) (kg/m${}^{2}$). Blood pressure (BP) was measured using a mercury sphygmomanometer under the following conditions: after a 10-min rest period, taken from the right arm, loose sleeves, non-fasting state, depleted bladder, and avoiding eating, drinking (except water), and smoking for at least one hr before the test.

We described ovulation disorder as the menstrual cycle duration in excess of 35 days or lack of menstrual cycle for more than three months (Oligo/anovulation) (16). We defined biochemical HA based on the cut-off set by Hashemi and co-workers (20). The FG-score ≥ 8 was specified as clinical HA (21). All participants (PCOS and controls) underwent abdominal ultrasonography. Ovaries containing 12 or more follicles measuring 2-9 mm in diameter and/or enlarged ovarian volume (> 10 mm${}^{3}$) on abdominal ultrasonography were considered to have a positive polycystic sonographic view (3).

MetS was recognized by the presence of three or more of the following risk factors based on the modified National Cholesterol Education Program Adult Treatment Panel III guidelines (22): fasting serum glucose (FBS) level of at least 100 mg/dl; fasting serum TG at least 150 mg/dl, serum High-density lipoprotein cholesterol (HDL-C) level < 50 mg/dl, systolic blood pressure (SBP) level of at least 130 mmHg, diastolic blood pressure (DBP) level of at least 85 mmHg, and waist circumference (WC) of at least 95 cm in Iranian race females (23).

Serologic hormonal and metabolic evaluations were performed between the 2nd and the 10th days of the menstrual cycle or on any day in amenorrheic condition. All samples were collected between 8 and 10 am. The specimens were drawn after overnight 12-hr fasting for the definition of plasma HDL-C, TG, and FBS. Blood samples were also drawn for assessment of SHBG and TT levels. FAI was computed by TT (nmol/L)/SHBG (nmol/L) ×100. TG and FBS were distinguished based on the Colorimetric-Enzymatic methods (glucose oxidase), and HDL-C was evaluated according to immunoinhibition methods, all of them by commercial kits (Pars Azmoon Inc., Tehran, Iran) using Auto-analyzer BT2000 device. Biochemical measurement of TT and SHBG levels was performed based on electro-chemiluminescence method (Roche Instr Kit, Germany) by Cobas E411 device.

### Ethical consideration

This study was approved by the Ethics Committee of Medical Sciences of Tarbiat Modares University (IRB # 525503). All women were informed about the project and fulfilled the written informed consent before participating in the study.

### Statistical analysis

Normal and non-normal quantitative variables were reported as Mean ± Standard Deviation (SD) and Median (interquartile range), respectively. Qualitative variables were presented as number (percentage). Primarily, the quantitative variables were checked for normality using the Kolmogorov-Smirnoff's (KS) test. One-way ANOVA was applied for the normal variables and Kruskal-Wallis (KW) test was used for the non-normal and ordinal variables. If there was a significant group effect, a pairwise comparison of the groups was performed using the Mann-Whitney's U test (MW). Then, Bonferroni's correction performance (p < 0.005) was considered significant. Qualitative variables were compared by Chi-square test and Fisher's exact test. In order to compare the non-normal and normal variables between the two PCOS groups with and without MetS, independent Samples *t* test and Mann-Whitney's test (MW) were used, respectively. The multiple linear regression analysis with the stepwise method was applied to evaluate the association between androgenic components (TT, FAI, and SHBG) as the independent variables and MetS components (SBP, DBP, TG, HDL, FBS, and WC) as the dependent variables in each group. Statistical significance was set at p < 0.05. Data were analyzed using the SPSS software (Statistical Package for the Social Sciences, SPSS Inc., Chicago, IL, USA), version 22.0.

## 3. Results

Table I gives some information about the Basic features of PCOS diagnosis in each group at the beginning of the study. According to Table II, there is no significant difference between the study groups in terms of age, BMI, physical activity, educational level, and socioeconomic status.

As shown in Table III, there is a significant difference between the groups in terms of MetS frequency, which is 17.1% (group H + P + O), 3% (group H + P), 2.5% (group O + P), 13.5% (group H + O), and 0.0% (control group). All PCOS subgroups had higher FBS compared to the control group. There was a statistically significant difference between phenotypes H + P + O, O + P, and H + O of PCOS and the control group in terms of HDL-C. Phenotypes H + P + O and H + O showed significant difference in SBP and DBP. Phenotype H + O and the control group showed a significant difference in SBP. There was a significant difference in DBP between phenotypes H + P and H + O.

In Table IV, the androgenic components of PCOS women with MetS (n = 14) are compared with those without MetS who were matched in terms of age and BMI (n = 28), regardless of phenotype classification. Since the numbers of PCOS women with MetS were only 14 people and it would be underpowered in comparison with 137 PCOS women without MetS, 28 age- and BMI-matched participants out of 137 PCOS women without MetS were selected and the androgenic components (TT, FAI, and SHBG) were compared between these two groups. TT and FAI were significantly higher in the PCOS group suffering from MetS. Nevertheless, there was no significant difference in the SHBG between the two groups. Table V gives the outcome of the stepwise linear regression analysis, including the TT, FAI, and SHBG as independent variables and MetS components (FBS, HDL-C, TG, WC, SBP, and DBP) as dependent variables in each group separately. In phenotype H + P + O, there is a statistically significant positive association between TG and TT, and a significant negative association between SBP and DBP with SHBG. In phenotype O + P, WC is inversely associated with SHBG. In phenotype H + O, FBS and TG are positively associated with FAI but HDL is inversely associated with FAI. Moreover, WC and DBP are positively associated with TT in phenotype H + O. However, no associations were detected between MetS components and TT, FAI, and SHBG in phenotype H + P and the control group (data are not shown).

**Table 1:** Basic features of PCOS diagnosis in each group at the beginning of the study


**Parameters**	**H + P + O group (n = 41)**	**H + P group (n = 33)**	**O + P group (n = 40)**	**H + O group (n = 37)**	**Control group (n = 31)**
**TT (nmol/L),**	2.68 ± 2.05	1.70 ± 1.45	1.17 ± 0.48	1.91 ± 1.40	0.77 ± 0.57
**FAI**	8.9 ± 7.7	4.0 ± 4.5	3.1 ± 2.9	5.5 ± 3.6	1.4 ± 1.5
**SHBG (nmol/L)**	41.5 ± 27.2	51.1 ± 28.3	50.7 ± 23.0	39.8 ± 20.2	76.3 ± 45.1
**FG-score**	9.3 ± 2.0	9.1 ± 1.5	2.5 ± 2.2	8.7 ± 1.5	2.1 ± 1.5
**Ovarian follicle counts**	14.4 ± 0.9	14.6 ± 1.0	13.9 ± 1.9	5.7 ± 3.1	5.6 ± 2.4
TT: Total testosterone; FAI: Free androgen index; SHBG: Sex hormone-binding globulin; FG-score: Ferriman-Gallwey score Data are given as Mean ± SD					

**Table 2:** Comparison of the demographic parameters at the beginning of the study


**Parameters**	**H + P + O group (n = 41)**	**H + P group (n = 33)**	**O + P group (n = 40)**	**H + O group (n = 37)**	**Control group (n = 31)**	**P-value**
**Age (years), median (IQR)**	28.0 (7.5)	27.0 (6.5)	26.0 (9.0)	28.0 (9.0)	29.0 (8.0)	0.09*
**BMI (Kg/m${}^{2}$), mean ± SD**	25.4 ± 5.2	25·3 ± 5·3	25.0 ± 4.2	25.0 ± 3.9	24.9 ± 4.8	0.99**
**Physical activity (exercise frequency), N (%)**						
**None**	23 (56.1)	20 (60.6)	25 (62.5)	25 (67.6)	20 (64.5)	
**1-2 times/week**	6 (14.6)	6 (18.2)	7 (17.5)	5 (13.5)	5 (16.1)	
**≥ 3 times/week**	12 (29.3)	7 (21.2)	8 (20.0)	7 (18.9)	6 (19.4)	0.90***
**Educational level, N (%)**						
**Basic school**	4 (9.8)	3 (9.1)	4 (10.0)	2 (5.4)	2 (6.5)		
**High school**	9 (22.0)	5 (15.2)	10 (25.0)	16 (43.2)	14 (45.2)	
**Bachelor**	19 (46.3)	16 (48.5)	20 (50.0)	15 (40.5)	10 (32.3)	
**MSc & PhD**	9 (22.0)	9 (27.3)	6 (15.0)	4 (10.8)	5 (16.1)	0.32***
**Socioeconomic status, N (%)**						
**First level (weak)**	16 (39)	23 (69.7)	23 (57.5)	21 (56.8)	17 (54.8)		
**Second level (average)**	16 (39)	5 (15.2)	11 (27.5)	7 (18.9)	7 (22.6)		
**Third level (good)**	9 (22)	5 (15.2)	6 (15)	9 (24.3)	7 (22.6)	0.20***
BMI: Body mass index *P-value refers to the Kruskal-Wallis test; **P-value refers to the One-way ANOVA; ***P-values refer to Chi-square test Statistical significance was set at p < 0.05.						

**Table 3:** Comparison of MetS and its components between PCOS subgroups and control group


	**H + P + O group${}^{A}$ (n = 41)**	**H + P group${}^{B}$ (n = 33)**	**O + P group${}^{C}$ (n = 40)**	**H + O group${}^{D}$ (n = 37)**	**Control (n = 31)**	P-value*	**pair wise comparison; MW, P-value**
**MetS (+) n (%)**	7 (17.1)	1 (3)	1 (2.5)	5 (13.5)	0 (0.0)	0.01	**-**
**FBS, median (IQR)**	91.0 (7.5)	88.0 (10.5)	87.0 (10.7)	90.0 (16.5)	79.0 (17.0)	<0.001	A & Control, p < 0.001 B & Control, p = 0.001 C & Control, p = 0.005 D & Control, p = 0.004
**HDL-C, median (IQR)**	45.0 (16.5)	52.0 (15.0)	51.5 (13.2)	49.0 (13.0)	56.0 (10.0)	<0.001	A & Control, p < 0.001 C & Control, p = 0.002 D & Control, p < 0.001
**TG, median (IQR)**	126.0 (93.5)	94.0 (86.5)	90.5 (77.0)	99.0 (91.5)	85.0 (70.0)	0.40	-
**WC, median (IQR)**	85.0 (14.5)	81.0 (11.0)	82.0 (11.5)	84.0 (12.7)	81.0 (15.0)	0.65	-
**SBP, median (IQR)**	110.0 (16.0)	110.0 (13.0)	114.5 (10.0)	118.0 (16.0)	110.0 (13.0)	<0.001	A & D, p = 0.003 D & Control, p = 0.004
**DBP, median (IQR)**	68.0 (27.5)	70.0 (15.0)	74.5 (20.0)	79.0 (10.0)	70.0 (12.0)	0.01	A & D, p = 0.003 B & D, p = 0.003
MetS: Metabolic syndrome; FBS: Fasting blood sugar; HDL-C: High-density lipoprotein cholesterol; TG: Triglyceride; WC: Waist circumference; SBP: Systolic blood pressure; DBP: Diastolic blood pressure; MW: Mann-Whitney's U test *P-values refer to the Kruskal-Wallis test followed by appropriate post hoc test (Mann-Whitney U test) (p < 0.005 was considered significant for multiple comparisons based on Bonferroni correction); p-value for the variable MetS refers to the Fisher's exact test (p < 0.05 was considered significant) Significant results are shown in the table exclusively							

**Table 4:** Comparison of androgenic components between PCOS women with and without MetS


	**PCOS**		**P-value**
**Androgenic components**	**MetS (+) n = 14**	**MetS (-) n = 28**	
	**TT (nmol/L)**	3.42 ± 3.02	1.09 ± 0.61	< 0.001*
**FAI**	9.91 ± 9.57	3.85 ± 3.53	0.022**
**SHBG (nmol/L)**	37.67 ± 13.47	39.88 ± 19.60	0.708*
PCOS: Polycystic ovary syndrome; MetS: Metabolic syndrome; TT: Total testosterone; FAI: Free androgen index; SHBG: Sex hormone binding globulin Data presented as Mean ± SD. *P-value refers to the independent samples *t* test; **P-value refers to the Mann-Whitney's U procedure.			

**Table 5:** Stepwise multiple linear regression analysis of androgenic predictive factors on MetS components in 182 Iranian women


**Significant predictors**	**Group**	**Dependent variables**	**B**	**SE**	**P-value**	**Adjusted R${}^{2}$**
	A	TG	45.215	16.471	0.009	0.14
	WC	8.719	4.122	0.040	0.08
**TT**	D	DBP	14.769	6.566	0.031	0.10
			FBS	0.901	0.382	0.026	0.11
		HDL-C	-1.338	0.395	0.002	0.22
**FAI**	D	TG	7.312	2.015	0.001	0.25
			SBP	-0.138	0.058	0.020	0.10
			A	DBP	-0.171	0.071	0.022	0.10
**SHBG**	C	WC		-0.160	0.058	0.009	0.14
	The models included TT, FAI, and SHBG as predictors, dependent variables included MetS components. Except for the independent variables shown, there were no significant relationships between the other variables FBS: Fasting blood sugar; HDL-C: High-density lipoprotein cholesterol; TG: Triglyceride; WC: Waist circumference; SBP: Systolic blood pressure; DBP: Diastolic blood pressure; TT: Total testosterone; FAI: Free androgen index; SHBG: Sex hormone-binding globulin; B: Unstandardized coefficient; SE: Standard error H.P.O. A: H + P + O; B: H + P; C: O + P; D: H + O Statistical significance was set at p < 0.05						

## 4. Discussion

This study compared the MetS ingredients between different phenotypes of PCOS and normal participants in a sample of Iranian women. The results showed that the frequency of MetS is significantly higher in all phenotypes of PCOS compared with the control group. It is necessary to mention that non-hyperandrogenic phenotype of PCOS (phenotype O + P) has the lowest frequency of MetS among all PCOS women. Hyperandrogenism, as the most eminent diagnostic indicator of PCOS, and its indices substantially depend on age, race, ethnicity and body weight. Several studies have so far been conducted to assess the prevalence of MetS among PCOS women with various phenotypes in different races. In concordance with our results, Kavardzhikova and colleagues illustrated significant differences in anthropometric, hormonal and metabolic components between the different phenotypes of PCOS (24). Also, Goverde and co-workers reported that the prevalence of metabolic derangements and IR varies between different phenotypes of PCOS. They described that hyperandrogenic phenotypes of PCOS have a higher prevalence of MetS and IR (25). In addition, the results of Jamil and colleagues study are relatively in accordance with our findings although they reported the highest prevalence of MetS in phenotype H + O, which can be justified by high BMI (26). In this study, hyperandrogenic subgroups of PCOS (subgroup H + P + O had the most frequency of MetS) showed a higher frequency of MetS. Furthermore, we omitted confounder BMI and age by matching between the participants. Conversely, Hosseinpanah and co-workers observed no difference in the MetS components of different PCOS phenotypes (27). The feasible explanation behind the aforementioned contradiction can be slight differences in the inclusion criteria, genetic factors, lifestyle characteristics, and dietary habits. Moreover, the studied groups in their research had a considerable difference in terms of participants' number. In accordance with our findings, Diamanti-Kandarakis and colleagues have also considered hyperandrogenemia as a contributing factor in MetS development among PCOS women (28). It is obvious that subgroup H + P + O, as a classic form of PCOS, has worse status than the others. Therefore, it seems that the highest prevalence of MetS in this phenotype of PCOS is justifiable. In agreement with the present study results, Pehlivanov and co-workers indicated that phenotype H + P + O PCOS patients (full Rotterdam) were more obese, with considerably higher expressed hyperandrogenemia and IR compared with other phenotypes of PCOS (29).

We also found that among all the components of MetS, FBS, HDL, SBP, and DBP showed significant differences between the different groups. However, FBS alone displayed significantly higher amounts in all phenotypes of PCOS than the control group. Also, HDL values in all phenotypes of PCOS except phenotype H + P were lower than in the control group. Similar to our study, Zahiri and colleagues reported a significant difference in MetS components between different phenotypes of PCOS and the control group (13). Thus far, several studies have been conducted in order to investigate MetS prevalence and metabolic profile changes in four phenotypes of PCOS. Zhang and co-workers reported 28.5%, 25.5%, 8.3%, and 7.2% of MetS frequency in H + P + O, H + O, H + P, and O + P phenotypes of PCOS, respectively, as compared to 3.5% in controls (19). Additionally, they found that some metabolic components (total cholesterol, low-density lipoprotein, TG, and HDL) were significantly different between the phenotypes of PCOS and control group. The results of the present study confirmed the highest frequency of MetS in the H + P + O group. However, the prevalence of MetS in all phenotypes of PCOS and controls was lower in the present study. In this study, the majority of participants (in the control group and PCOS subgroups) were very young and had lower BMI. This is a very important group of women to target for further intervention and prevention strategies in the development of MetS. Furthermore, all individuals who were consuming antihypertensive, lipid, and glucose-lowering drugs were not included in the study. Also, different diagnostic criteria (International Diabetic Federation) of MetS were used in their study. Low prevalence of MetS in four phenotypes of PCOS and zero prevalence in controls in this study can be caused by these points. Also, Kar and colleagues evaluated the metabolic profile of the four Rotterdam PCOS phenotypes. Contrary to the present study results, they found no significant difference in metabolic components except for WC between groups (14). Absence of the age- and BMI-matched control group to PCOS phenotypes in their study can justify this contradiction. The significant differences observed in the metabolic components in the present study were often between the PCOS subgroups and the control group. Furthermore, there was a notable difference in the sample size in the four groups in Kar's study (14). phenotype P + H included only 0.9% (n = 4), and by contrast, phenotype H + P + O comprised 65.6% (n = 269) out of all participants that could reduce the study power. Tripathy and co-workers reported that WC was significantly greater in phenotypes H + P + O and H + O (15). They also represented the increased serum levels of HDL-C in H + P + O and H + O as compared to phenotype O + P and controls. Additionally, significant increased level of TG in phenotypes H + P + O and H + O compared to other groups was displayed. MetS prevalence was 33.6%, 36.7%, 24.4%, 11.6%, and 10.1% in phenotype H + P + O, H + O, H + P, O + P, and controls, respectively. The method used in the mentioned study had contained some subtle differences: Not matching between groups in terms of age and BMI and striking difference in sample size between four phenotypes with each other and controls. These observed differences can be attributed to match the participants in terms of BMI. Moreover, observed differences in results of the various studies may be justified by the impression of genetic diversity between various ethnic populations and different environmental factors and also by delicate differences in the selected methods and inclusion criteria used by each of them.

It is worth noting that numerous studies have examined the impact of hyperandrogenism on the MetS components separately though the majority of them were performed in a group of PCOS without phenotype consideration (30, 31). As a novel work, we analyzed the effect of androgenic predictive factors on MetS components in all phenotypes of PCOS separately. Our results demonstrated the undesirable impact of androgenic ingredients on MetS components. Furthermore, we found a significantly higher level of TT and FAI in PCOS women with MetS compared with those without MetS. Kar and co-workers also compared the androgenic components values (TT, free testosterone, and SHBG) between the two PCOS groups with and without MetS. They found no significant difference in mentioned components between the groups (14). This result could likely be different if BMI and age matching were applied between the two groups. Since, FAI is an androgenic component derived from a formula (TT (nmol/L)/SHBG (nmol/L) × 100), this component is affected by TT and SHBG. A significant increase in FAI in PCOS patients with MetS compared with those without MetS (as a result of the present study) can be affected by a significant increase in TT in these individuals. Indeed, it can be stated that TT is more relevant to MetS, and a significant increase in this component has led to an increase in FAI in these patients. The higher FAI-relevant P-value can be influenced by SHBG which does not show any significant difference between the two groups. Moreover, it seems that TT could be a more potent predictor of MetS components than FAI and SHBG. Based on the results of linear regression analysis, the effectiveness of TT on MetS components was more potent compared with FAI and SHBG. Some of the previous studies evidenced that hyperandrogenemia has adverse effects on abnormal lipid profile (32, 33).

The findings of Dreno B and co-workers study have indicated that there is a correlation between androgenic components and the number of MetS components (34). The results of Fruzzetti and co-workers study, in favour of this relationship, showed a significant positive correlation between free testosterone and FAI with the number of MetS components, whereas SHBG had a significant negative correlation with MetS components (35). This study has three valuable strengths: 1) Strict inclusion criteria of the study reduced greatly the confounding factors; 2) Other factors, which can influence our results, were removed by matching; 3) The study investigated the association of hyperandrogenic elements with MetS components separately in all phenotypes of PCOS. However, this is a cross-sectional study, and since the cross-sectional designs limit the data interpretation to the correlation association between variables, it is suggested to carry out more cohort or case-control studies in future to achieve a causal relationship. Moreover, the participants in the present study were very young cohort average below 27 years old and their mean BMI was around 25. Maybe if their age and MBI were higher, it would be more possible for the frequency of MetS to be more. It is recommended to conduct a long-term study (cohort) in this field to get more accurate results.

## 5. Conclusion

The findings of the present study confirm the presence of metabolic changes in PCOS patients. It was also revealed that there are significant differences in the metabolic profiles among different PCOS phenotypes. According to our results, hyperandrogenic components per se (TT, FAI, and SHBG) could be predictors of metabolic disorders and thus increased risk of MetS. It is notable that our results were not suggesting a routine investigation of androgenic parameters as a part of the evaluation of PCOS patients in order to prevent from MetS. This topic needs longer investigations (cohort) with more sample sizes. We propose that MetS screening and a metabolic inspection could be lucrative to prevent MetS and its related complications in PCOS women, at least in Iranian women.

##  Conflict of Interest

The authors declare that there are no conflicts of interest.
